# Quantum renewal processes

**DOI:** 10.1038/s41598-020-62260-z

**Published:** 2020-03-27

**Authors:** Bassano Vacchini

**Affiliations:** 1 Dipartimento di Fisica “Aldo Pontremoli”, Università degli Studi di Milano, Via Celoria 16, Milan, 20133 Italy; 2 INFN, Sezione di Milano, Via Celoria 16, Milan, 20133 Italy

**Keywords:** Quantum mechanics, Theoretical physics

## Abstract

We introduce a general construction of master equations with memory kernel whose solutions are given by completely positive trace-preserving maps. These dynamics going beyond the Lindblad paradigm are obtained with reference to classical renewal processes, so that they are termed quantum renewal processes. They can be described by means of semigroup dynamics interrupted by jumps, separated by independently distributed time intervals, following suitable waiting time distributions. In this framework, one can further introduce modified processes, in which the first few events follow different distributions. A crucial role, marking an important difference with respect to the classical case, is played by operator ordering. Indeed, for the same choice of basic quantum transformations, different quantum dynamics arise. In particular, for the case of modified processes, it is natural to consider the time inverted operator ordering, in which the last few events are distributed differently.

## Introduction

The proper description of the dynamics of a quantum system in many cases of relevance calls for taking into account all other degrees of freedom, typically called environmental, which might affect its time evolution. In such cases one speaks of the dynamics of an open quantum system^1,2^. Indeed, closed systems, strictly isolated from any other degree of freedom over any time scale, are rather an exception. When dealing with open quantum systems, a generally valid evolution equation such as the Schrödinger equation for isolated systems is not known. A class of dynamics which has proven to be of great relevance is given by semigroups, which break in a natural way the reversibility inherent in the unitary evolution. These semigroup evolutions are obtained as solution of master equations whose structure has been fully characterized^3,4^ and is typically called Lindblad form. They provide the natural quantum counterpart of classical Markovian semigroups, and indeed has been first introduced in view of this analogy^5^. As a result evolutions of Lindblad type has proven a reference result for all situations in which a Markovian approach can be considered, and memory effects can be neglected. This is however often not the case, e.g. due to strong coupling or low temperatures. The characterization of more general evolution equations, which might take into account non-Markovian effects, is therefore a pressing issue. In this direction one can consider two main possible approaches, i.e. either time-local master equations or integro-differential ones involving a memory kernel (MK). The key difficulty in both approaches, which has not yet found a general solution, is determining the conditions on the structure of the master equation warranting trace preservation and complete positivity of the solutions. Memory kernels warranting this property are usually termed legitimate. Various efforts have been done in this direction, leading to partial results both with reference to equations in time-local form^6–12^, as well as to equations in time non local form^13–24^.

In this paper we will provide a derivation of classes of legitimate MK, relying on the analogy with classical stochastic processes. Obtaining MK master equations has shown to be a daunting task, but it appears that very large classes can be introduced and connected to a very simple physical interpretation as well as a natural probabilistic interpretation. The quantum processes arising as solution of these equations are connected to quantum versions of classical renewal processes and modified renewal processes, and are characterized by the fact that they provide a piecewise continuous dynamics in which continuous time evolutions of semigroup type are interrupted by jumps described as completely positive trace preserving (CPT) transformations. A renewal process is a counting process describing the stochastic realization of jumps^25,26^. The times among subsequent jumps are given by independent identically distributed random variables characterized by an arbitrary common waiting time distributions (WTD). A generalization of renewal processes is given by so-called modified renewal processes, in which the first few time intervals follow a different distribution with respect to the subsequent ones. In the classical context renewal processes are a powerful tool for the description of random walks and transport in complex systems, as well as in reliability theory (see e.g.^27,28^ and references therein). The starting point of this analysis will be a suitable correspondence rule from classical commuting quantities to operators, in the same spirit of^23,24^. Two new aspects are considered for the first time in this work, thus allowing to significantly enlarge the class of known quantum MK warranting as solutions legitimate dynamics: the introduction of modified quantum renewal processes and the consideration of inverse time operator ordering, which still leads to well-defined dynamics. This approach encompasses simple examples already considered in the literature^15,20,29–32^ and puts them within a more general theory. It thus opens the way for considering more general dynamics, e.g. in the framework of collision models, which have recently attracted a lot of interest providing a powerful tool to address issues in quantum thermometry, quantum thermodynamics, quantum optics, quantum entanglement and quantum non-Markovianity^33–43^. Indeed, collision models are naturally introduced as dynamics characterized by a sequence of collisions or jumps. The variety of such models in the dependence on the jump operators as well as features and possible interactions between the environmental components has been extensively analyzed^33,38,43–47^, while little has been done to investigate the relevance of the distribution in time of the interaction events. This theoretical proposal provides a groundwork for the study of these effects, allowing in particular to deal with situations in which a selection of collision events have to be treated differently.

The paper is organized as follows. We first outline the basic formalism and discuss previous approaches for the treatment of MK. We further introduce the notion of quantum renewal process, later investigating the different processes arising by considering modified WTD and the inverse time order in the allocation of jumps respectively. We then consider a few simple examples, pointing to possible developments in the final discussion.

## Results

### Memory kernels and generalized master equations

Let us first recall the general framework. We say that the dynamics of a system is described by a MK master equation if the time dependent statistical operator *ρ*(*t*) describing the statistics of observations on the system obeys

1 $$\frac{{\rm{d}}}{{\rm{d}}t}\rho (t)=\,{\int }_{0}^{t}{\rm{d}}\tau {\mathscr{W}}(t-\tau )[\rho (\tau )]+{\mathscr{I}}(t)[\rho (0)],$$

where the superoperator ${\mathscr{W}}(t)$ is called MK, while the superoperator ${\mathscr{I}}(t)$ is usually termed inhomogeneous contribution. We stress the fact that the term memory is used because one is faced with an integral equation with respect to the operator-valued variable *ρ*(*t*). This is not directly related to a notion of memory in the quantum dynamics, as possibly captured by the different recently introduced notions of quantum non-Markovianity^48–50^. Since the master equation Eq. (1) is meant to describe the evolution in time of a statistical operator, the corresponding solutions should comply with two basic requirements, namely preservation of trace and positivity of the state. Assuming that this dynamics arises as a consequence of the interaction of the system of interest with some environment, then also complete positivity has to be asked for. Introducing the linear transformation ${\mathscr{D}}(t)$ giving the time evolution

2 $$\rho (t)={\mathscr{D}}(t)[\rho (0)],$$

and therefore obeying

3 $$\frac{{\rm{d}}}{{\rm{d}}t}{\mathscr{D}}(t)=\,{\int }_{0}^{t}{\rm{d}}\tau {\mathscr{W}}(t-\tau ){\mathscr{D}}(\tau )+{\mathscr{I}}(t)$$

with the initial condition ${\mathscr{D}}(0)={\mathbb{1}}$, these requirements correspond to take ${\{{\mathscr{D}}(t)\}}_{t\in {{\mathbb{R}}}_{+}}$ as a collection of CPT maps.

The quest for introducing MK, and possibly corresponding inhomogeneous contributions, which lead to well-defined quantum transformation going beyond the standard Lindblad dynamics, has proven to be quite hard, though some reference results have been obtained, formulating either sufficient or necessary conditions on the superoperator expressions. In particular, making reference to the theory of semi-Markov processes, a class of non-Markovian classical stochastic processes, it has proven possible to obtain a large collection of legitimate quantum MK. To obtain such dynamics, which have been termed quantum semi-Markov processes, one considers a quantum master equation of the form Eq. (3) with a vanishing inhomogeneous term, namely

4 $$\frac{{\rm{d}}}{{\rm{d}}t}\rho (t)=\,{\int }_{0}^{t}{\rm{d}}\tau {\mathscr{K}}(t-\tau )[\rho (\tau )],$$

with MK superoperators given by ${\hat{{\mathscr{K}}}}_{l}(u)=u+\hat{g{\mathscr{G}}}{(u)}^{-1}(\,\hat{f{\mathcal{F}}}(u)-\,{\mathbb{1}})$ and ${\widehat{{\mathscr{K}}}}_{r}(u)=u+(\,\widehat{f{\mathscr{F}}}(u)-\,{\mathbb{1}})\widehat{g{\mathscr{G}}}{(u)}^{-1}$, where the indexes *l*, *r* denote left and right respectively, in view of operator ordering, while the hat is used to denote the Laplace transform according to

5 $$\hat{h}(u)={\int }_{0}^{{\rm{\infty }}}{\rm{d}}t\,h(t){{\rm{e}}}^{-ut}.$$

Depending on the context *h*(*t*) will denote either a function or a time dependent collection of operators. The motivation for this choice of kernels comes from the analogy with classical semi-Markov processes, as we shall argue below making reference to Eqs. (9) and (10). The kernels are built in terms of the Laplace transform of the operators $f(t){\mathscr{F}}(t)$ and $g(t){\mathscr{G}}(t)$, where *f*(*t*) is a WTD, and *g*(*t*) the corresponding survival probability. Namely *f*(*t*) is a probability distribution over the positive time axis and

6 $$g(t)=1-{\int }_{0}^{t}{\rm{d}}\tau f(\tau )$$

gives the probability of no jumps up to time *t*^25,26^. These MK master equations have solutions given by CPT transformations if ${\{{\mathscr{F}}(t)\}}_{t\in {{\mathbb{R}}}_{+}}$ and ${\{{\mathscr{G}}(t)\}}_{t\in {{\mathbb{R}}}_{+}}$ are arbitrary collection of CPT maps, with the only further constraint ${\mathscr{G}}(0)={\mathbb{1}}$. The associated time evolutions can be shown to be given by the collections of maps

7 $${\widehat{{\mathscr{D}}}}_{l}(u)=\widehat{g{\mathscr{G}}}(u){\left({\mathbb{1}}-\widehat{f{\mathscr{F}}}(u)\right)}^{-1}$$

and

8 $${\widehat{{\mathscr{D}}}}_{r}(u)={({\mathbb{1}}-\widehat{f{\mathscr{F}}}(u))}^{-1}\widehat{g{\mathscr{G}}}(u),$$

respectively. Though different approaches have been considered to obtain MK falling in this class^15,23,51,52^, the possibly simplest starting point to recover and understand these results is to make contact with the generalized master equation for the transition probability *T*_*nm*_(*t*) of a semi-Markov process^53–56^ which reads

9 $$\frac{{\rm{d}}}{{\rm{dt}}\,}{T}_{nm}(t)={\int }_{0}^{t}d\tau \sum _{k}[{w}_{nk}(t-\tau ){T}_{km}(\tau )-{w}_{kn}(t-\tau ){T}_{nm}(\tau )],$$

where *T*_*nm*_(*t*) provides the probability to reach site *n* at time *t* given that one starts from an arbitrary but fixed site *m* at time *t* = 0. Indeed, a semi-Markov process describes the time evolution of a classical system which can jump among different sites according to fixed probabilities, the time elapsing between subsequent jumps being described by a collection of independent and identically distributed random variables, which might depend on the considered site. The process is specified by a so-called semi-Markov matrix, a time dependent matrix whose entries provide the probability density to jump between two sites in a given time. For the special case in which the semi-Markov matrix is given by a stochastic matrix times an exponential WTD, one recovers a classical Markovian jump process. In all other cases the classical process is non-Markovian. The semi-Markov matrix determines the MK *w*_*nk*_(*t*) appearing in Eq. (9), which in Laplace transform reads ${\widehat{w}}_{nk}(u)={\widehat{g}}_{n}(u){\pi }_{nk}{\widehat{f}}_{k}(u)$/${\widehat{g}}_{k}(u)$. Here *π*_*nk*_ are the elements of the stochastic matrix whose entries are the jump probabilities between sites, while *f*_*n*_(*t*) provides the WTD at site *n*, namely the probability distribution for the time elapsing before the next jump takes place. The function *g*_*n*_(*t*) is the corresponding survival probability, given by ${g}_{n}(t)=1-{\int }_{0}^{t}d\tau {f}_{n}(\tau )$. In Laplace transform the solution of the generalized master equation Eq. (9) reads

10 $${\widehat{T}}_{nm}(u)=\sum _{k}{\left({\mathbb{1}}-\pi \widehat{f}(u)\right)}_{nk}^{-1}{\widehat{g}}_{k}(u){\widehat{T}}_{km}(0).$$

The quantum maps Eqs. (7), (8) and related quantum MK ${\widehat{{\mathscr{K}}}}_{l,r}(u)$ can thus be obtained by using the following correspondence rule between functions and operator-valued expressions

11 $$f(t)\to f(t){\mathscr{F}}(t),\quad g(t)\to g(t){\mathscr{G}}(t)$$

together with a choice of operator ordering, which we have encoded in the *l*, *r* index. The relevance of operator ordering, rooted in non-commutativity of quantum transformations, brings with itself the fact that the very same classical kernel can lead to different quantum kernels. This dependence on ordering is due to the appearance of the maps ${\mathscr{F}}(t)$ and ${\mathscr{G}}(t)$, which describe the time evolution of the quantum system in between jumps. The effect of the stochastic matrix *π*, which in the quantum description is naturally replaced by a CPT map, has been reabsorbed in the collection ${\{{\mathscr{F}}(t)\}}_{t\in {{\mathbb{R}}}_{+}}$, which upon composition with a fixed CPT map still provides a time dependent collection of CPT maps.

In this framework one has two basic results. On the one hand one obtains a characterization of a very large class of legitimate quantum kernels; on the other hand the resulting dynamics can be naturally described as a piecewise quantum dynamics, in which continuous in time quantum evolutions are reset at given times or interrupted by jumps.

The obtained dynamics, for specific choices of the involved collections of maps and CPT transformations, has been shown to be connected to physical models. More specifically maps of the form Eq. (7) provide the mathematical description for the dynamics of the micromaser^57–59^, while transformations as in Eq. (8) correspond to classes of collision models with memory^20,31,60^.

### Quantum renewal processes

We first consider the case in which the collection of CPT maps ${\{{\mathscr{F}}(t)\}}_{t\in {{\mathbb{R}}}_{+}}$ and ${\{{\mathscr{G}}(t)\}}_{t\in {{\mathbb{R}}}_{+}}$ can be obtained as quantum dynamical semigroups composed with fixed jump transformations. In this setting we fix the dynamics taking place in between jumps and concentrate on the effect of the jump transformations and the time elapsed in between jumps, so that by analogy with classical renewal processes^25,26^ it is natural to call such dynamics quantum renewal processes. We therefore take the collections ${\{{\mathscr{F}}(t)\}}_{t\in {{\mathbb{R}}}_{+}}$ of maps to be of the form

12 $${\mathscr{F}}(t)={\mathscr{E}}{{\rm{e}}}^{{\mathcal{L}}t}{\mathscr{J}},$$

with ${\mathscr{L}}$ an arbitrary generator in Lindblad form, while ${\mathscr{E}}$ and ${\mathscr{J}}$ are arbitrary CPT maps, together with ${\mathscr{G}}(t)={{\rm{e}}}^{{\mathscr{M}}t}$, with ${\mathscr{M}}$ still in Lindblad form, according to the relation ${\mathscr{G}}(0)={\mathbb{1}}$. For each of these choices of time dependent transformation we have two distinct kernels, arising due to *l* and *r* operator ordering. The relevance of this ordering will be discussed in detail in Sec., while here we focus on the *l* case. Exploiting the fact that multiplication by an exponential function in Laplace transform goes over to translation, as shown in the Methods’s section we have the kernel

13 $${\widehat{{\mathscr{K}}}}_{l}(u)={\mathscr{M}}+({\mathscr{E}}\widehat{f}(u-{\mathscr{L}}){\mathscr{J}}-\widehat{f}(u-{\mathscr{M}}))\widehat{g}{(u-{\mathscr{M}})}^{-1}.$$

Considering the corresponding expression of the evolution map ${\widehat{{\mathscr{D}}}}_{l}(u)=\widehat{g}\left(u-{\mathscr{M}}\right){\left({\mathbb{1}}-{\mathscr{E}}\widehat{f}(u-{\mathscr{L}}){\mathscr{J}}\right)}^{-1}$ in time, so that multiplication goes over to convolution, expanding the Neumann series we therefore obtain

14 $$\rho (t)=g(t){{\rm{e}}}^{{\mathscr{M}}t}[\rho (0)]+\mathop{\sum }\limits_{n=1}^{{\rm{\infty }}}{\int }_{0}^{t}{\rm{d}}{t}_{n}\ldots {\int }_{0}^{{t}_{2}}{\rm{d}}{t}_{1}\,g(t-{t}_{n}){{\rm{e}}}^{{\mathscr{M}}(t-{t}_{n})}{\mathscr{E}}f({t}_{n}-{t}_{n-1}){{\rm{e}}}^{{\mathscr{L}}({t}_{n}-{t}_{n-1})}\ldots {\mathscr{J}}{\mathscr{E}}f({t}_{1}){{\rm{e}}}^{{\mathscr{L}}{t}_{1}}{\mathscr{J}}[\rho (0)].$$

**Figure 1:**
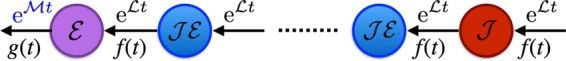
Scheme of a time evolution described by a quantum renewal process. In between jumps the time evolution is given by a semigroup, possibly a different one for the last time interval. The jumps are described by CPT transformations which might differ in both initial and final application.

The evolution is thus described as a piecewise dynamics, interrupted by jumps, in which initial, final and intermediate transformations can be different, as shown in Fig. 1.

At the same time trace preservation is generally warranted by the fact that

15 $$g({t}_{})+\mathop{\sum }\limits_{n=1}^{{\rm{\infty }}}{\int }_{0}^{t}{\rm{d}}{t}_{n}\ldots {\int }_{0}^{{t}_{2}}{\rm{d}}{t}_{1}\,g(t-{t}_{n})f({t}_{n}-{t}_{n-1})\ldots f({t}_{2}-{t}_{1})f({t}_{1})=1,$$

as follows from the theory of renewal processes^25,26^. In particular one can consider a different semigroup evolution for the time before the last jump. All these dynamics share the fact of being describable as a combination of semigroup dynamics over independent identically distributed time intervals. Non-commutativity implies that at variance with the classical case, for quantum renewal processes also the time at which the jumps take place affects the dynamics. Special examples of this framework have been previously considered in the literature, as one of the first examples of legitimate MK^15,61^. While we stress the fact that even in the simplified case in which one of the transformations is trivial, i.e. either ${\mathscr{E}}$ or ${\mathscr{J}}$ is the identity transformation, Eq. (12) combined with the choice of ordering leads to four distinct quantum dynamics, it is of interest to work out in more detail a special case, to show the connection with the standard Markovian semigroup dynamics. To determine the dynamics we have to specify different quantities, namely the generators ${\mathscr{L}}$ and ${\mathscr{M}}$, the quantum channels ${\mathscr{E}}$ and ${\mathscr{J}}$, as well as the WTD *f*(*t*). Let us take ${\mathscr{M}}={\mathscr{L}}$, ${\mathscr{J}}={\mathbb{1}}$ and *f*(*t*) = *λ*e^−*λt*^, that is we consider an exponential waiting time, which in the classical case leads to a Markov renewal process, namely a Poisson process. As shown in the Methods’s section the MK takes the form ${\mathscr{K}}(t)=\delta (t)[{\mathscr{L}}+\lambda ({\mathscr{E}}-{\mathbb{1}})]$, corresponding to a semigroup dynamics given by the sum of two generators

16 $${\mathscr{D}}(t)={{\rm{e}}}^{[{\mathscr{L}}+\lambda ({\mathscr{E}}-{\mathbb{1}})]t}.$$

One can now exploit the relation ${(u-(A+B))}^{-1}={(u-A)}^{-1}{\left(1-B{(u-A)}^{-1}\right)}^{-1}$ valid for two arbitrary operators *A* and *B* and leading to the Dyson expansion

17 $${{\rm{e}}}^{(A+B)t}={{\rm{e}}}^{At}+\mathop{\sum }\limits_{n=1}^{\infty }{\int }_{0}^{t}{\rm{d}}{t}_{n}\ldots {\int }_{0}^{{t}_{2}}{\rm{d}}{t}_{1}{{\rm{e}}}^{A(t-{t}_{n})}B{{\rm{e}}}^{A({t}_{n}-{t}_{n-1})}\ldots B{{\rm{e}}}^{A{t}_{1}},$$

for the two possible splittings of the argument of the exponential in Eq. (16). The apparently most natural choice is $A={\mathscr{L}}$ and $B=\lambda ({\mathscr{E}}-{\mathbb{1}})$, leading to

18 $${\mathscr{D}}(t)={{\rm{e}}}^{{\mathscr{L}}t}+\mathop{\sum }\limits_{n=1}^{\infty }{\int }_{0}^{t}{\rm{d}}{t}_{n}\ldots {\int }_{0}^{{t}_{2}}{\rm{d}}{t}_{1}{{\rm{e}}}^{{\mathscr{L}}(t-{t}_{n})}\lambda ({\mathscr{E}}-{\mathbb{1}})\ldots {{\rm{e}}}^{{\mathscr{L}}({t}_{2}-{t}_{1})}\lambda ({\mathscr{E}}-{\mathbb{1}}){{\rm{e}}}^{{\mathscr{L}}{t}_{1}}$$

to be compared with the alternative choice $A={\mathscr{L}}-\lambda \,{\mathbb{1}}$ and $B=\lambda \,{\mathscr{E}}$ leading to

19 $${\mathscr{D}}(t)={{\rm{e}}}^{-\lambda t}{{\rm{e}}}^{{\mathscr{L}}t}+\mathop{\sum }\limits_{n=1}^{\infty }{\int }_{0}^{t}{\rm{d}}{t}_{n}\ldots {\int }_{0}^{{t}_{2}}{\rm{d}}{t}_{1}{{\rm{e}}}^{-\lambda ({t}_{}-{t}_{n})}{{\rm{e}}}^{{\mathscr{L}}(t-{t}_{n})}{\mathscr{E}}\ldots {\mathscr{E}}\lambda {{\rm{e}}}^{-\lambda ({t}_{2}-{t}_{1})}{{\rm{e}}}^{{\mathscr{L}}({t}_{2}-{t}_{1})}{\mathscr{E}}\lambda {{\rm{e}}}^{-\lambda {t}_{1}}{{\rm{e}}}^{{\mathscr{L}}{t}_{1}}.$$

Both representations are exact. It now immediately appears that Eq. (19), arising from a mixture with positive coefficients of Lindblad generators, is a special case of Eq. (14) for the choice of an exponential WTD with rate *λ*, together with ${\mathscr{J}}={\mathbb{1}}$ and ${\mathscr{M}}={\mathscr{L}}$. Also the equivalent expression Eq. (18) can be written in a way which allows to connect to a generic WTD. Indeed, a renewal process is uniquely determined from its WTD or equivalently its renewal density *S*(*t*), also known as sprinkling distribution, arising as solution of the renewal equation $S(t)=f(t)+{\int }_{0}^{t}{\rm{d}}\tau f(t-\tau )S(\tau )$. For the case of a memoryless exponential waiting time the sprinkling distribution, which gives the probability density to have a jump at the given time, neglecting all previous jumps, is a constant function, simply given by the rate *λ*. Indeed, one can check that for ${\mathscr{J}}={\mathbb{1}}$ and ${\mathscr{M}}={\mathscr{L}}$ the original time evolution Eq. (14) allows for the two equivalent expressions

20 $$\begin{array}{lll}\rho (t) & = & g(t){{\rm{e}}}^{{\mathscr{L}}t}[\rho (0)]+\mathop{\sum }\limits_{n=1}^{\infty }{\int }_{0}^{t}{\rm{d}}{t}_{n}\ldots {\int }_{0}^{{t}_{2}}{\rm{d}}{t}_{1}\\  &  & \times g(t-{t}_{n}){{\rm{e}}}^{{\mathscr{L}}(t-{t}_{n})}{\mathscr{E}}f({t}_{n}-{t}_{n-1}){{\rm{e}}}^{{\mathscr{L}}({t}_{n}-{t}_{n-1})}\ldots {\mathscr{E}}f({t}_{1}){{\rm{e}}}^{{\mathscr{L}}{t}_{1}}[\rho (0)]\\ \end{array}$$

21 $$\begin{array}{lll} & = & {{\rm{e}}}^{{\mathscr{L}}t}[\rho (0)]+\mathop{\sum }\limits_{n=1}^{\infty }{\int }_{0}^{t}{\rm{d}}{t}_{n}\ldots {\int }_{0}^{{t}_{2}}{\rm{d}}{t}_{1}\\  &  & \times {{\rm{e}}}^{{\mathscr{L}}(t-{t}_{n})}({\mathscr{E}}-{\mathbb{1}})S({t}_{n}-{t}_{n-1}){{\rm{e}}}^{{\mathscr{L}}({t}_{n}-{t}_{n-1})}\ldots ({\mathscr{E}}-{\mathbb{1}})S({t}_{1}){{\rm{e}}}^{{\mathscr{L}}{t}_{1}}[\rho (0)],\end{array}$$

as follows from the operator identity

22 $$\widehat{g}(u-{\mathscr{L}})\frac{1}{{\mathbb{1}}-{\mathscr{E}}\widehat{f}(u-{\mathscr{L}})}=\frac{1}{u-{\mathscr{L}}}\frac{1}{{\mathbb{1}}-({\mathscr{E}}-{\mathbb{1}}) {\hat{S}} (u-{\mathscr{L}})},$$

proven in the Methods’s section.

The expression Eq. (20) of the time evolved state can be interpreted as a sum of contributions corresponding to a piecewise dynamics with a different number of intermediate jumps. During the jumps described by the CPT map ${\mathscr{E}}$ the state evolves according to a semigroup dynamics determined by ${\mathscr{L}}$ for a time interval (*t*_*n*_ − *t*_*n*−1_) fixed by the waiting time *f*(*t*_*n*_ − *t*_*n*−1_). Each term in the sum provides a contribution to the trace of *ρ*(*t*), corresponding to subcollections characterized by a given number of jumps. In a complementary way expression Eq. (21) is the sum of a purely semigroup dynamics together with terms determined by the repeated appearance of contributions of the form $({\mathscr{E}}-{\mathbb{1}})S({t}_{n}-{t}_{n-1})$. The latter can be interpreted saying that with a probability density given by the sprinkling distribution *S*(*t*_*n*_ − *t*_*n*−1_) the time evolved contribution is replaced by another in which an additional ${\mathscr{E}}$ transformation has acted upon, hence the operator $({\mathscr{E}}-{\mathbb{1}})$. In between these transformations one still has a semigroup dynamics.

The different MK and related time evolutions considered above differ by the choice of generators ${\mathscr{L}}$ and ${\mathscr{M}}$, the choice of channels ${\mathscr{E}}$ and ${\mathscr{J}}$, as well as WTD *f*(*t*). The appearance of *f*(*t*) warrants trace preservation, while details of the dynamics are determined by the different operators. We have however always made reference to the kernel ${{\mathscr{K}}}_{l}(t)$ corresponding to one choice of operator ordering, that is a specific order in time in which events takes place. This marks an important difference with respect to the classical case, which we shall put in evidence later on, after considering modified renewal processes.

### Modified quantum renewal processes

We now derive another class of quantum renewal processes, which can be named modified renewal processes since in analogy with the classical case they correspond to a situation in which the WTD characterizing the first *k* intervals differ from the following ones. Starting from the identity Eq. (15), which warranted trace preservation in the previous examples, moving to the Laplace transform and exploiting $u\widehat{g}(u)=\left(1-\widehat{f}(u)\right)$, one obtains

23 $$\frac{1}{u}={\widehat{g}}_{1}(u)+\ldots +{\widehat{g}}_{k}(u){\widehat{f}}_{k-1}(u)\ldots {\widehat{f}}_{1}(u)+\widehat{g}(u)\frac{1}{1-\widehat{f}(u)}{\widehat{f}}_{k}(u)\ldots {\widehat{f}}_{1}(u),$$

describing the normalization condition for the situation in which the first *k* jumps have a different waiting time. Here again *g*_*k*_(*t*) denotes the survival probability associated to the WTD *f*_*k*_(*t*) according to ${g}_{k}(t)=1-{\int }_{0}^{t}d\tau {f}_{k}(\tau )$. A quantum dynamics corresponding to such modified renewal processes can be obtained via the operator replacements

24 $${\widehat{f}}_{k}(u)\to {\widehat{f}}_{k}(u-{\mathscr{L}})\quad {\widehat{g}}_{k}(u)\to {\widehat{g}}_{k}(u-{\mathscr{L}}),$$

leading to the collection of CPT maps

25 $${\widehat{{\mathscr{D}}}}_{\overleftarrow{k}}(u)={\widehat{g}}_{1}(u-{\mathscr{L}})+\ldots +{\widehat{g}}_{k}(u-{\mathscr{L}}){\mathscr{E}}{\widehat{f}}_{k-1}(u-{\mathscr{L}})\ldots {\mathscr{E}}{\widehat{f}}_{1}(u-{\mathscr{L}})+\widehat{g}(u-{\mathscr{L}})\frac{1}{{\mathbb{1}}-{\mathscr{E}}\widehat{f}(u-{\mathscr{L}})}{\mathscr{E}}{\widehat{f}}_{k}(u-{\mathscr{L}})\ldots {\mathscr{E}}{\widehat{f}}_{1}(u-{\mathscr{L}}),$$

where the arrow appearing in the index denotes the natural time order from right to left in distinguishing the waiting times. The MK associated to these modified dynamics are quite involved, but it is natural to express them and the associated evolution equations making reference to the MK for the unmodified case, and considering the effect of the modified waiting times by means of inhomogeneous contributions to the equation. We therefore first introduce the unmodified dynamics

26 $${\widehat{{\mathscr{D}}}}_{\mathop{0}\limits^{\leftarrow}}(u)=\widehat{g}(u-{\mathscr{L}})\frac{1}{{\mathbb{1}}-{\mathscr{E}}\widehat{f}(u-{\mathscr{L}})},$$

so that according to Eq. (7) we have for the related kernel

27 $${\widehat{{\mathscr{K}}}}_{\mathop{0}\limits^{\leftarrow}}(u)={\mathscr{L}}+({\mathscr{E}}-{\mathbb{1}})\widehat{k}(u-{\mathscr{L}}),$$

where we have introduced the quantity

28 $$\widehat{k}(u)=\frac{\widehat{f}(u)}{\widehat{g}(u)},$$

which corresponds to the classical kernel associated to the renewal process^25,26^. Starting from the relation

29 $$u{\hat{{\mathscr{D}}}}_{\overleftarrow{k}}(u)-{\mathbb{1}}={\hat{{\mathscr{K}}}}_{\overleftarrow{0}}(u){\hat{{\mathscr{D}}}}_{\overleftarrow{k}}(u)+{\hat{{\mathscr{I}}}}_{\overleftarrow{k}}(u)$$

we obtain, as shown in the Methods’s section

30 $$\begin{array}{lll}{\widehat{{\mathscr{I}}}}_{\overleftarrow{k}}(u) & = & \left({\mathscr{E}}-{\mathbb{1}}\right)\left({ {\hat{S}} }_{1}(u-{\mathscr{L}})- {\hat{S}} (u-{\mathscr{L}})\right)\\  &  & +\left({\mathscr{E}}-{\mathbb{1}}\right)\left({ {\hat{S}} }_{2}(u-{\mathscr{L}})- {\hat{S}} (u-{\mathscr{L}})\right){\mathscr{E}}{\widehat{f}}_{1}(u-{\mathscr{L}})\\  &  & +\ldots \\  &  & ({\mathscr{E}}-{\mathbb{1}})\left({ {\hat{S}} }_{k}(u-{\mathscr{L}})- {\hat{S}} (u-{\mathscr{L}})\right){\mathscr{E}}{\widehat{f}}_{k-1}(u-{\mathscr{L}})\ldots {\mathscr{E}}{\widehat{f}}_{1}(u-{\mathscr{L}}),\end{array}$$

corresponding to the master equation

31 $$\begin{array}{lll}\frac{{\rm{d}}}{{\rm{dt}}\,}\rho (t) & = & {\mathscr{L}}[\rho (t)]+{\int }_{0}^{t}{\rm{d}}\tau ({\mathscr{E}}-{\mathbb{1}}){{\rm{e}}}^{{\mathscr{L}}(t-\tau )}k(t-\tau )\rho (\tau )\\  &  & +({\mathscr{E}}-{\mathbb{1}}){{\rm{e}}}^{{\mathscr{L}}t}({S}_{1}(t)-S(t))[\rho (0)]\\  &  & +\mathop{\sum }\limits_{r=2}^{k}{\int }_{0}^{t}{\rm{d}}{t}_{r-1}\ldots {\int }_{0}^{t}{\rm{d}}{t}_{1}({\mathscr{E}}-{\mathbb{1}}){{\rm{e}}}^{{\mathscr{L}}(t-{t}_{r-1})}({S}_{r}(t-{t}_{r-1})-S(t-{t}_{r-1}))\\  &  & \times {\mathscr{E}}{f}_{r-1}({t}_{r-1}-{t}_{r-2}){{\rm{e}}}^{{\mathscr{L}}({t}_{r-1}-{t}_{r-2})}\ldots {\mathscr{E}}{f}_{1}({t}_{1}){{\rm{e}}}^{{\mathscr{L}}{t}_{1}}[\rho (0)].\end{array}$$

The master equation can also be written in the form Eq. (4), with a MK that can be compactly expressed in terms of the inhomogeneous contribution Eq. (30)

32 $$\begin{array}{lll}{\widehat{{\mathscr{K}}}}_{\overleftarrow{k}}(u) & = & {\mathscr{L}}+\frac{1}{{\mathbb{1}}+{\widehat{{\mathscr{I}}}}_{\overleftarrow{k}}(u)}\{({\mathscr{E}}-{\mathbb{1}})\widehat{k}(u-{\mathscr{L}})+{\widehat{{\mathscr{I}}}}_{\overleftarrow{k}}(u)(u-{\mathscr{L}})\}\end{array}.$$

In particular, if only the first time interval is different from the others one recovers for the kernel the slightly more compact expression

33 $${\widehat{{\mathscr{K}}}}_{\overleftarrow{1}}(u)={\mathscr{L}}+\frac{1}{{\mathbb{1}}-({\mathscr{E}}-{\mathbb{1}})\left( {\hat{S}} (u-{\mathscr{L}})-{ {\hat{S}} }_{1}(u-{\mathscr{L}})\right)}({\mathscr{E}}-{\mathbb{1}}){\widehat{k}}_{1}(u-{\mathscr{L}}).$$

This provides a straightforward generalization of one of the first results about quantum MK^15,61^, and for ${\mathscr{L}}=0$, that is neglecting the intermediate time evolution, leads to an evolution of the form $\rho (t)={\widehat{{\mathscr{D}}}}_{\overleftarrow{1}}(u)[\rho (0)]={\sum }_{n=1}^{\infty }\,{p}_{1}(n,t){{\mathscr{E}}}^{n}[\rho (0)]$, where *p*_1_(*n*, *t*) provide the probabilities to have *n* jumps up to time *t* for the modified process. An alternative representation of the master equation, which can be more easily connected to^15,61^ is obtained by considering the reference kernel Eq. (27) together with the inhomogeneous contribution

34 $${\widehat{{\mathscr{I}}}}_{\overleftarrow{1}}(u)=({\mathscr{E}}-{\mathbb{1}})\left({ {\hat{S}} }_{1}(u-{\mathscr{L}})- {\hat{S}} (u-{\mathscr{L}})\right)$$

confirming the result obtained in^24,32^. The expression of the master equation Eq. (31) shows that the inhomogeneous contribution, due to the presence of different WTD characterizing the first jumps, is directly dependent on the initial condition, as in the standard derivation of MK master equations within projection operator techniques^1^. It is worth noticing that inhomogeneous terms also appear in other approaches considering dependence of the dynamics on the preparation time^62^.

### Inverse time operator ordering

In the previous analysis we have highlighted the relevance of having non commuting quantities which, even for a fixed sequence of events, lead to different evolution equations and different dynamics, at variance with the classical case. We now put into evidence another peculiar quantum feature, arising from the fact that when replacing the relation Eq. (23) with the operator valued Eq. (25) the ordering in time of the events becomes crucial and instead of the situation in which the first *k* waiting time intervals have a different distribution, one can consider the situation in which the last *k* are characterized in a different way, corresponding to

35 $${\widehat{{\mathscr{D}}}}_{\overrightarrow{k}}(u)={\widehat{g}}_{1}(u-{\mathscr{L}})+\ldots +{\widehat{f}}_{1}(u-{\mathscr{L}}){\mathscr{E}}\ldots {\widehat{f}}_{n-1}(u-{\mathscr{L}}){\mathscr{E}}{\widehat{g}}_{n}(u-{\mathscr{L}})+{\widehat{f}}_{1}(u-{\mathscr{L}}){\mathscr{E}}\ldots {\widehat{f}}_{n}(u-{\mathscr{L}}){\mathscr{E}}\frac{1}{{\mathbb{1}}-\widehat{f}(u-{\mathscr{L}}){\mathscr{E}}}\widehat{g}(u-{\mathscr{L}}).$$

**Figure 2:**
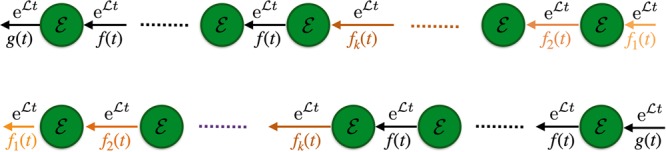
Schematic representation of the two possible time evolution described by a modified quantum renewal process. In both dynamics the jump transformations take place after independent time intervals, which are however not all identically distributed. In particular one can consider situations in which the first *k* waiting times (top), or the last *k* ones (bottom) follow different distributions.

The relevant ordering describing the actual dynamics will depend on the considered physical situation. A special realization of this feature has been considered in^24^ comparing a micromaser dynamics with a class of collision models. The index now denotes the inverse time ordering, from left to right, and one can notice that ${\widehat{{\mathscr{D}}}}_{\overrightarrow{k}}(u)={\widehat{{\mathscr{D}}}}_{\overleftarrow{k}}^{T}(u)$, where we have used the symbol *T* to denote the inverse operator ordering, i.e. ${({A}_{1}\ldots {A}_{n})}^{T}={A}_{n}\ldots {A}_{1}$, since indeed this evolution map can be obtained from Eq. (25) by inverting the operator ordering. The two situations described by a modified quantum renewal process together with a choice of operator ordering is schematically shown in Fig. 2.

The reference dynamics is now given by

36 $${\widehat{{\mathscr{D}}}}_{\overrightarrow{0}}(u)=\frac{1}{{\mathbb{1}}-{\mathscr{E}}\widehat{f}(u-{\mathscr{L}})}\widehat{g}(u-{\mathscr{L}}),$$

with kernel

37 $${\widehat{{\mathscr{K}}}}_{\overrightarrow{0}}(u)={\mathscr{L}}+\widehat{k}(u-{\mathscr{L}})({\mathscr{E}}-{\mathbb{1}}),$$

again connected to Eqs. (26) and (27) respectively by inverting the operator ordering. The master equation providing a closed evolution equation for the dynamics given by Eq. (35) can be written as

38 $$\frac{{\rm{d}}}{{\rm{d}}t}\rho (t)=\,{\int }_{0}^{t}{\rm{d}}\tau {{\mathscr{K}}}_{\overrightarrow{k}}(t-\tau )[\rho (\tau )],$$

with a kernel simply given by ${\widehat{{\mathscr{K}}}}_{\overrightarrow{k}}(u)={\widehat{{\mathscr{K}}}}_{\overleftarrow{k}}^{T}(u)$. The major difference in considering as different the last *k* waiting times is best appreciated writing the master equation equivalent to Eq. (38) but expressed using the reference MK Eq. (37) and a inhomogeneous contribution

39 $$\frac{{\rm{d}}}{{\rm{d}}t}\rho (t)=\,{\int }_{0}^{t}{\rm{d}}\tau {{\mathscr{K}}}_{\overrightarrow{0}}(t-\tau )[\rho (\tau )]+{{\mathscr{I}}}_{\overrightarrow{k}}(t)[\rho (0)],$$

where now the inhomogeneous term takes the natural but involved expression

40 $${\widehat{{\mathscr{I}}}}_{\overrightarrow{k}}(u)={\widehat{{\mathscr{D}}}}_{\overrightarrow{0}}{(u)}^{-1}{\widehat{{\mathscr{I}}}}_{\overleftarrow{k}}^{T}(u){\widehat{{\mathscr{D}}}}_{\overrightarrow{0}}(u).$$

At variance with the expression Eq. (30) appearing in the master equation Eq. (31), where the effect of having a modified renewal process is expressed as a simple correction in time, here the inhomogeneous correction is obtained convoluting the time reversed inhomogeneous term with free propagators forward and backward in time.

### Examples

In order to exemplify the introduced formalism and to point out the different dynamical behavior that can arise as a consequence of operator ordering, we consider a few examples. The obtained class of legitimate MK, and therefore CPT dynamics, depends both on the choice of jump transformations and intermediate time evolution maps, as well as on the considered WTD characterizing the different time intervals. Here we will focus in particular on the comparison between a quantum renewal process and its modified counterpart, as well as on the different dynamics arising by considering the same sequence of events but in a different time operator ordering.

Let us first consider the difference between Eq. (25), describing a dynamics in which the first *k* time intervals are characterized by a different WTD, and its unmodified counterpart Eq. (26). To this aim, despite the fact that the obtained results are not constrained to finite dimensional Hilbert spaces, we consider for the sake of simplicity a two-level system. This allows in particular to have a simple matrix representation of the different maps involved. Indeed, for a fixed basis of operators in the Hilbert space, which we take to be given by the identity and the Pauli matrices apart from a normalization factor, each map ${\mathscr{A}}[\,\cdot \,]$ can be represented by a four dimensional matrix with entries ${A}_{ij}=\frac{1}{2}{\rm{Tr}}\,({\sigma }_{i}{\mathscr{A}}[{\sigma }_{j}])$, with *i*, *j* = 0, 1, 2, 3. In this representation in particular map composition goes over to matrix multiplication^9,63^, so that expressions of the form Eqs. (25) and (26) can be easily evaluated. We take as reference dynamics a semigroup evolution describing exponential dephasing and damping according to

41 $${{\rm{e}}}^{{\mathscr{L}}t}[{\sigma }_{i}]={{\rm{e}}}^{-{\lambda }_{i}t}{\sigma }_{i}$$

for *i* = 1, 2, 3, while assuming an intermediate transformation ${\mathscr{E}}$ obtained by considering an amplitude damping channel, possibly composed with a dephasing transformation. It is obviously crucial to consider non commuting transformations, i.e. to fulfil the requirement $[{\mathscr{E}},{\mathscr{L}}]\ne 0$. According to Eq. (41) one can further exploit the following matrix representation for maps given by functions of $u-{\mathscr{L}}$

42 $$ {\hat{h}} (u-{\mathscr{L}})={\rm{diag}}\,\left( {\hat{h}} (u), {\hat{h}} (u+{\lambda }_{1}), {\hat{h}} (u+{\lambda }_{2}), {\hat{h}} (u+{\lambda }_{3})\right).$$

**Figure 3:**
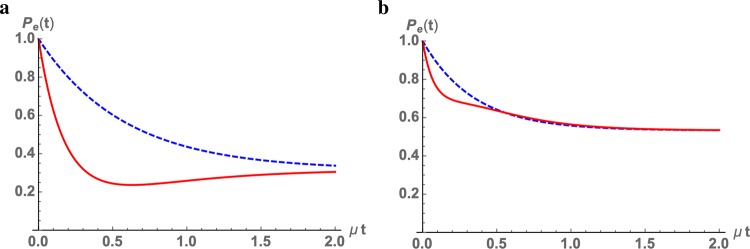
Behavior of the population of the excited state of a two-level system *P*_*e*_(*t*). In each panel we compare a quantum renewal process and a modified version of it. Panel (a) dynamics described by an exponential damping with rate *λ*_3_ = 1.1 in inverse units of time, intertwined with jumps described by a amplitude damping channel with parameter *γ* = 0.8^66^. The solid curve (red) corresponds to the modified quantum renewal process corresponding to Eq. (25), while the dashed curve (blue) to the unmodified one of Eq. (26). Here *k* = 3 so that for the modified process we have considered three exponential distributions with different rates in the ratio 1:7:5. The populations are plotted as function of *μt*, with *μ* the parameter characterizing the first WTD. Panel (b) dynamics with the same damping rate and WTD in the ratio 1:5:10, but jumps described by the composition of a Pauli channel with Kraus operator *σ*_*x*_ and an amplitude damping with parameter *γ* = 0.43.

The final information necessary in order to fix the structure of the dynamical map is given by the choice of WTD, which we take in the first instance as exponential, i.e. of the form *μ*e^−*μt*^, albeit with different rates *μ*. Such a WTD describes Poisson distributed events with rate *μ*. The difference in the obtained dynamics can be seen plotting the behavior in time of the population of the excited state, as shown in Fig. 3. In particular it can be seen how the modified process can lead to a non monotonic decrease of the population of the excited state.

**Figure 4:**
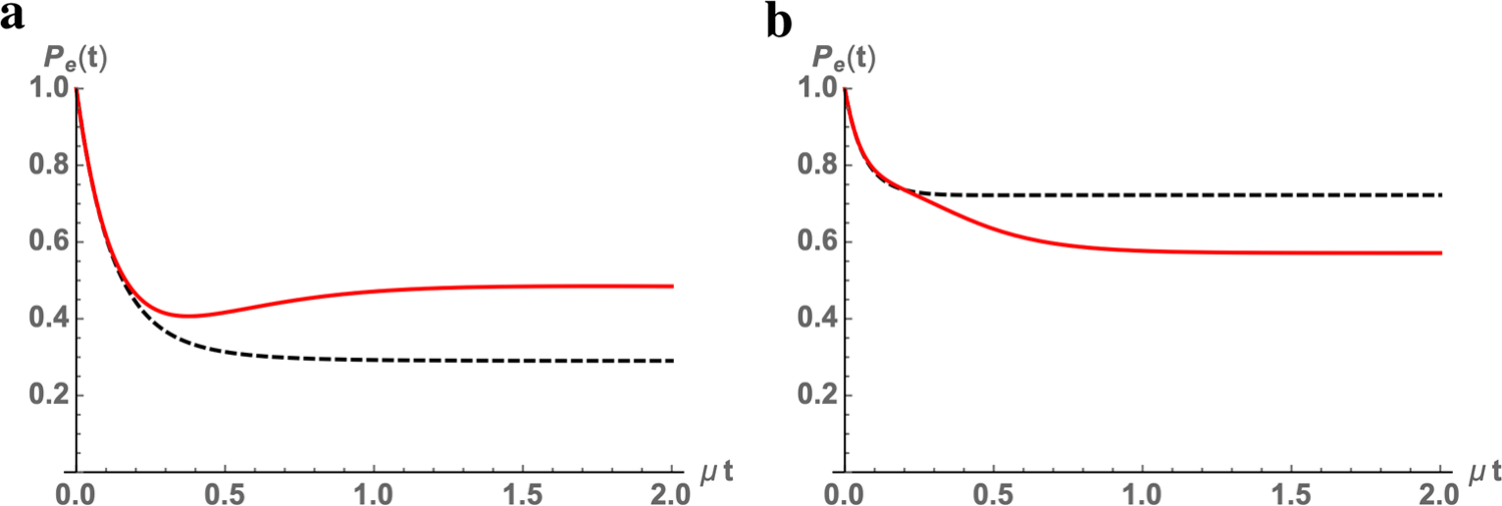
Time evolution of the occupation of the excited state *P*_*e*_(*t*). The compared evolutions only differ for the time ordering. In both panels we consider a dynamics described by an exponential damping with rate *λ*_3_ = 4 in inverse units of time. The process is characterized by four different exponential WTD, so that we have *k* = 4. In panel (a) their rates are in the ratio 1:5:.1:.5, while in panel (b) the ratio is given by 1:10:20:15. Jumps are described by an amplitude damping channel with parameter *γ* = 0.87^66^, further composed with a dephasing map in panel (b). The solid curves (red) correspond to the quantum renewal process ordered as in Eq. (25), while the dashed curves (black) to the process with inverse time ordering as in Eq. (35).

As a further illustration, we consider for the same system the situation in which the dynamics only differ for the operator ordering. That is we consider the distinct evolution maps Eqs. (25) and (35) for the same specification of the generator ${\mathscr{L}}$ determining the intermediate time evolution and the same channel describing the jumps, as well as WTD. To this aim we still consider the semigroup dynamics given by Eq. (41). The variety of possible different behavior is put into evidence in Fig. 4, where we have considered in the different panels quantum processes only differing for the choice of channels and rates of the involved waiting times. The inverse operator ordering corresponds to the solid curves and typically brings in an important modification of the dynamics before a stationary situation is reached.

The considered situations only provide an illustrative example of the different behavior that can arise considering solutions of the quantum dynamics that we have introduced as a quantum version of classical renewal processes. Actual implementations will depend on dimensionality and details of the considered system, as well as on the feature of the interactions determining its reduced dynamics. It is important however to stress that examples of realization of special cases in physical systems have already appeared in the physical literature, e.g. modified WTD in the micromaser dynamics^29,30,32^ or dynamics related to MK corresponding to different orderings in the treatment of non-Markovian collision models^20,31,64,65^.

## Discussion

We have constructed a new, large class of quantum MK, possibly including inhomogeneous terms, which provide master equations whose solutions are indeed CPT transformations. Though the construction of legitimate MK, providing more general dynamics than the standard Lindblad one, has proven to be a very difficult task^13,16,19^, as we have shown a natural and fruitful viewpoint is to make reference to classical non-Markovian processes. In this framework we have considered a convenient strategy for introducing a class of quantum transformations that have been termed quantum renewal processes, due to the fact that they are built starting from classical renewal processes. The basic ingredients of the construction are indeed a collection of distributions over the time axis and a CPT channel describing quantum transformations taking place in between an intermediate semigroup evolution, after intervals dictated by the waiting time. This framework allows to recover and unify in a compact and elegant way previous results on MK master equations, as well as it provides classes of new legitimate MK.

In the construction one can consider and put into evidence two important aspects. In the first instance one can deal with modified processes, so that the time intervals between subsequent quantum transformations are independent but not identically distributed. Furthermore, for each legitimate MK one can consider another distinct kernel determined by an inverted operator ordering and physically corresponding to a reversed sequence of interaction events. This new MK still leads to a well-defined dynamics, and from a mathematical point of view it is essentially obtained by transposition. In all these dynamics a crucial role is played by the typical quantum feature of non-commutativity, bringing with itself the relevant role played by operator ordering. Simple examples have been provided, showing that indeed the interplay of these different features can lead to a wide variety of behavior, further recalling that special cases of this general framework have appeared in the description of physical systems^30,31^.

Despite significantly enlarging the known classes of MK leading to well-defined reduced time evolutions, this contribution leaves open the question about the most general characterization of such kernels. In particular one might wonder what is the most general form of piecewise dynamics leading to closed evolution equations in integral forms, and to what extent these kind of dynamics can exhibit non-Markovian effects. An open interesting issue is whether these non-Markovian dynamics can be embedded in Markovian models by a suitable enlargement of degrees of freedom. These questions naturally call for future investigations.

## Methods

### Derivation of quantum renewal process with intermediate evolution map ${\mathscr{F}}(t)={\mathscr{E}}{{\rm{e}}}^{{\mathscr{L}}t}{\mathscr{J}}$

In order to obtain the expression Eq. (13) for the kernel, leading to Eq. (14) for the evolved state, we start from Eq. (4), which in Laplace transform leads to the following general relationship between map and MK

43 $$\widehat{{\mathscr{K}}}(u)=u-\widehat{{\mathscr{D}}}{(u)}^{-1},$$

as well as the relationship

44 $$\frac{1}{\widehat{g}(u-{\mathscr{M}})}=(u-{\mathscr{M}})+\widehat{k}(u-{\mathscr{M}}),$$

which follows from Eq. (28) together with the Laplace transform expression of the survival probability $u\widehat{g}(u)=\left(1-\widehat{f}(u)\right)$. Assuming now expression Eq. (7) for the time evolution map, with a collection of intermediate time evolution maps ${\{{\mathscr{F}}(t)\}}_{t\in {{\mathbb{R}}}_{+}}$ given by Eq. (12) together with ${\mathscr{G}}(t)={{\rm{e}}}^{{\mathscr{M}}t}$, we recover the expression anticipated in Eq. (13)

45 $$\begin{array}{lll}{\widehat{{\mathscr{K}}}}_{l}(u) & = & u-\left({\mathbb{1}}-{\mathscr{E}}\widehat{f}(u-{\mathscr{L}}){\mathscr{J}}\right)\widehat{g}{(u-{\mathscr{M}})}^{-1}\\  & = & {\mathscr{M}}-\widehat{k}(u-{\mathscr{M}})+\left({\mathscr{E}}\widehat{f}(u-{\mathscr{L}}){\mathscr{J}}\right)\widehat{g}{(u-{\mathscr{M}})}^{-1}\\  & = & {\mathscr{M}}+\left({\mathscr{E}}\widehat{f}(u-{\mathscr{L}}){\mathscr{J}}-\widehat{f}(u-{\mathscr{M}})\right)\widehat{g}{(u-{\mathscr{M}})}^{-1}.\end{array}$$

For the case of exponential WTD *f*(*t*) = *λ*e^−*λt*^, we have the simple relationship $\widehat{f}(u)=\lambda \widehat{g}(u)$. In particular this implies that if ${\mathscr{J}}$ becomes the trivial transformation, ${\mathscr{J}}={\mathbb{1}}$, and the generators describing the time evolution in the first time interval and the subsequent ones do coincide, i.e. ${\mathscr{M}}={\mathscr{L}}$, then we are left with

46 $$\begin{array}{lll}\widehat{{\mathscr{K}}}(u) & = & {\mathscr{L}}+({\mathscr{E}}\widehat{f}(u-{\mathscr{L}})-\widehat{f}(u-{\mathscr{L}}))\widehat{g}{(u-{\mathscr{L}})}^{-1}\\  & = & {\mathscr{L}}+\lambda ({\mathscr{E}}-{\mathbb{1}}),\end{array}$$

and therefore in the time domain ${\mathscr{K}}(t)=\delta (t)[{\mathscr{L}}+\lambda ({\mathscr{E}}-{\mathbb{1}})]$, leading to the memoryless evolution equation Eq. (16).

### Proof of the operator identity Eq. (22)

In order to prove the operator identity Eq. (22) we start from the defining equation for the sprinkling distribution or renewal density associated to a renewal process^25,26^, namely

47 $$S(t)=f(t)+{\int }_{0}^{t}{\rm{d}}\tau f(t-\tau )S(\tau ),$$

leading in Laplace transform to the relation

48 $$ {\hat{S}} (u)=\frac{\widehat{f}(u)}{1-\widehat{f}(u)}.$$

Starting from the expression of the Laplace transform of the survival probability we then have the following chain of operator identities

49 $$\begin{array}{lll}\widehat{g}(u-{\mathscr{L}})\frac{1}{{\mathbb{1}}-{\mathscr{E}}\widehat{f}(u-{\mathscr{L}})} & = & \frac{{\mathbb{1}}-\widehat{f}(u-{\mathscr{L}})}{u-{\mathscr{L}}}\frac{1}{{\mathbb{1}}-{\mathscr{E}}\widehat{f}(u-{\mathscr{L}})}\\  & = & \frac{{\mathbb{1}}-\widehat{f}(u-{\mathscr{L}})}{u-{\mathscr{L}}}{\left({\mathbb{1}}-\widehat{f}(u-{\mathscr{L}})-({\mathscr{E}}-{\mathbb{1}})\widehat{f}(u-{\mathscr{L}})\right)}^{-1}\\  & = & \frac{{\mathbb{1}}-\widehat{f}(u-{\mathscr{L}})}{u-{\mathscr{L}}}{\left(\left({\mathbb{1}}-({\mathscr{E}}-{\mathbb{1}})\frac{\widehat{f}(u-{\mathscr{L}})}{{\mathbb{1}}-\widehat{f}(u-{\mathscr{L}})}\right)\left({\mathbb{1}}-\widehat{f}(u-{\mathscr{L}})\right)\right)}^{-1}\\  & = & \frac{1}{u-{\mathscr{L}}}{\left({\mathbb{1}}-({\mathscr{E}}-{\mathbb{1}})\frac{\widehat{f}(u-{\mathscr{L}})}{{\mathbb{1}}-\widehat{f}(u-{\mathscr{L}})}\right)}^{-1}\\  & = & \frac{1}{u-{\mathscr{L}}}\frac{1}{{\mathbb{1}}-({\mathscr{E}}-{\mathbb{1}}) {\hat{S}} (u-{\mathscr{L}})},\end{array}$$

leading to Eq. (22).

### Memory kernel master equations with homogeneous contribution

We now prove that the master equation, for the case of a modified quantum renewal process with the first *k* time intervals following a different distribution, can be expressed making reference to the MK for the unmodified case together with inhomogeneous contributions of the form Eq. (30). To this aim let us start from the general expression of MK master equation with inhomogeneous term given by Eq. (3), which in Laplace transform reads

50 $$\widehat{{\mathscr{D}}}(u)={\left(u-\widehat{{\mathscr{W}}}(u)\right)}^{-1}\left({\mathbb{1}}+\widehat{{\mathscr{I}}}(u)\right).$$

We now want to identify the inhomogeneous term for an evolution map given by Eq. (25), taking as reference kernel ${\widehat{{\mathscr{K}}}}_{\overleftarrow{0}}(u)$ as in Eq. (27), so that ${\left(u-{\widehat{{\mathscr{K}}}}_{\overleftarrow{0}}(u)\right)}^{-1}$ according to Eq. (43) corresponds to the unmodified dynamics Eq. (26) and we are left with

51 $${\widehat{{\mathscr{D}}}}_{k}(u)=\widehat{g}(u-{\mathscr{L}})\frac{1}{{\mathbb{1}}-{\mathscr{E}}\widehat{f}(u-{\mathscr{L}})}\left({\mathbb{1}}+{\widehat{{\mathscr{I}}}}_{k}(u)\right).$$

Considering in the first instance *k* = 1 we obtain the identity

52 $${\widehat{g}}_{1}(u-{\mathscr{L}})+\widehat{g}(u-{\mathscr{L}})\frac{1}{{\mathbb{1}}-{\mathscr{E}}\widehat{f}(u-{\mathscr{L}})}{\mathscr{E}}{\widehat{f}}_{1}(u-{\mathscr{L}})=\widehat{g}(u-{\mathscr{L}})\frac{1}{{\mathbb{1}}-{\mathscr{E}}\widehat{f}(u-{\mathscr{L}})}\left({\mathbb{1}}+{\widehat{{\mathscr{I}}}}_{1}(u)\right).$$

We now recall that $u{\widehat{g}}_{1}(u)=(1-{\widehat{f}}_{1}(u))$, while the sprinkling distribution for a modified process obeys

53 $${S}_{1}(t)={f}_{1}(t)+{\int }_{0}^{t}{\rm{d}}\tau f(t-\tau ){S}_{1}(\tau ),$$

so that

54 $${ {\hat{S}} }_{1}(u)=\frac{{\widehat{f}}_{1}(u)}{1-\widehat{f}(u)}.$$

We have in particular the relation

55 $${\widehat{g}}_{1}(u-{\mathscr{L}})=\widehat{g}(u-{\mathscr{L}})\left({\mathbb{1}}+ {\hat{S}} (u-{\mathscr{L}})-{ {\hat{S}} }_{1}(u-{\mathscr{L}})\right),$$

allowing to write the l.h.s. of Eq. (52) in the form

56 $$\widehat{g}(u-{\mathscr{L}})\left[{\mathbb{1}}+ {\hat{S}} (u-{\mathscr{L}})-{ {\hat{S}} }_{1}(u-{\mathscr{L}})+\frac{1}{{\mathbb{1}}-{\mathscr{E}}\widehat{f}(u-{\mathscr{L}})}{\mathscr{E}}{\widehat{f}}_{1}(u-{\mathscr{L}})\right],$$

so that Eq. (52) becomes equivalent to

57 $$\left({\mathbb{1}}-{\mathscr{E}}\widehat{f}(u-{\mathscr{L}})\right)\left({\mathbb{1}}+ {\hat{S}} (u-{\mathscr{L}})-{ {\hat{S}} }_{1}(u-{\mathscr{L}})+\frac{1}{{\mathbb{1}}-{\mathscr{E}}\widehat{f}(u-{\mathscr{L}})}{\mathscr{E}}{\widehat{f}}_{1}(u-{\mathscr{L}})\right)=({\mathbb{1}}+{\widehat{{\mathscr{I}}}}_{1}(u)).$$

This in turn leads to

58 $${\widehat{{\mathscr{I}}}}_{1}(u)=({\mathbb{1}}-{\mathscr{E}}\widehat{f}(u-{\mathscr{L}}))( {\hat{S}} (u-{\mathscr{L}})-{ {\hat{S}} }_{1}(u-{\mathscr{L}}))+{\mathscr{E}}({\widehat{f}}_{1}(u-{\mathscr{L}})-\widehat{f}(u-{\mathscr{L}}))$$

but according to Eq. (54) we have

59 $${\widehat{f}}_{1}(u-{\mathscr{L}})-\widehat{f}(u-{\mathscr{L}})=({\mathbb{1}}-\widehat{f}(u-{\mathscr{L}}))({ {\hat{S}} }_{1}(u-{\mathscr{L}})- {\hat{S}} (u-{\mathscr{L}}))$$

and therefore finally

60 $${\widehat{{\mathscr{I}}}}_{1}(u)=({\mathscr{E}}-{\mathbb{1}})({ {\hat{S}} }_{1}(u-{\mathscr{L}})- {\hat{S}} (u-{\mathscr{L}})).$$

To prove the relation in the general case we proceed by induction, omitting the common argument $u-{\mathscr{L}}$. We consider the case *k* + 1, which due to the expression of the evolution map corresponds to

61 $$\left({\mathbb{1}}-{\mathscr{E}}\widehat{f}\right)\left({\mathbb{1}}+ {\hat{S}} -{ {\hat{S}} }_{1}\right)+\ldots +({\mathbb{1}}-{\mathscr{E}}\widehat{f})({\mathbb{1}}+ {\hat{S}} -{ {\hat{S}} }_{k+1}){\mathscr{E}}{\widehat{f}}_{k}\ldots {\mathscr{E}}{\widehat{f}}_{1}+{\mathscr{E}}{\widehat{f}}_{k+1}\ldots {\mathscr{E}}{\widehat{f}}_{1}={\mathbb{1}}+{\widehat{{\mathscr{I}}}}_{k+1}(u)$$

but assuming assume ${\widehat{{\mathscr{I}}}}_{k}(u)$ to be of the form Eq. (30), adding and subtracting the term ${\mathscr{E}}{\widehat{f}}_{k}\ldots {\mathscr{E}}{\widehat{f}}_{1}$ we are left with

62 $$\begin{array}{lll}{\widehat{{\mathscr{I}}}}_{k+1}(u) & = & {\widehat{{\mathscr{I}}}}_{k}(u)+[({\mathbb{1}}-{\mathscr{E}}\widehat{f})({\mathbb{1}}+ {\hat{S}} -{ {\hat{S}} }_{k+1})-{\mathbb{1}}+{\mathscr{E}}{\widehat{f}}_{k+1}]{\mathscr{E}}{\widehat{f}}_{k}\ldots {\mathscr{E}}{\widehat{f}}_{1}\\  & = & {\widehat{{\mathscr{I}}}}_{k}(u)+[( {\hat{S}} -{ {\hat{S}} }_{k+1})-{\mathscr{E}}\widehat{f}( {\hat{S}} -{ {\hat{S}} }_{k+1})+{\mathscr{E}}({\widehat{f}}_{k+1}-\widehat{f})]{\mathscr{E}}{\widehat{f}}_{k}\ldots {\mathscr{E}}{\widehat{f}}_{1}\\  & = & {\widehat{{\mathscr{I}}}}_{k}(u)+({\mathscr{E}}-{\mathbb{1}})({ {\hat{S}} }_{k+1}- {\hat{S}} ){\mathscr{E}}{\widehat{f}}_{k}\ldots {\mathscr{E}}{\widehat{f}}_{1},\end{array}$$

where we have used again Eq. (59), thus obtaining the general result.
